# Evaluating Activation and Absence of Negative Effect: Gamification and Escape Rooms for Learning

**DOI:** 10.3390/ijerph17072224

**Published:** 2020-03-26

**Authors:** Jesús López-Belmonte, Adrian Segura-Robles, Arturo Fuentes-Cabrera, María Elena Parra-González

**Affiliations:** 1 Department of Didactics and School Organization, University of Granada, 18071 Granada, Spain; jesuslopez@ugr.es (J.L.-B.); arturofuentes@ugr.es (A.F.-C.); 2 Department of Research Methods and Diagnosis in Education, University of Granada, 18071 Granada, Spain; elenaparra@ugr.es

**Keywords:** educational innovation, active methodologies, gamification, escape room, psychosocial factors, positive effects, improvement of indicators

## Abstract

Innovation has allowed for and developed new ways of teaching and learning. Gamification is among the new training methodologies, which is a didactic approach based on the game structure with an attractive component for students. Within gamification, flipped learning and problem-based learning, escape rooms can be found as a technical aspect, which is focused on providing enigmas and tracks for the various educational content that students have assimilated through learning based on problem solving. The aim of this study is to identify how the use of gamification with the use of educational escape rooms affects activation and absence of a negative effect on students. 61 Master students of the Autonomous City of Ceuta participated in this case study. They were divided into three study groups (1 control group; 2 experimental groups) that followed different formative actions (control group—traditional; experimental groups—escape rooms). To achieve the objectives, a mixed research design based on quantitative and qualitative techniques was followed. The instrument used for data collection was the GAMEX (Gameful Experience Scale). The results reveal that the students who had taken a gamified formative action through escape rooms obtained better assessment results in the indicators concerning motivation, teamwork, commitment, activation, and absence of a negative effect on the learning process than those with the traditional methodology.

## 1. Introduction

Current education is involved in a whirlwind of methodological strategies and new ways of transmitting knowledge [1]. This is linked to the emergence of new educational tools and the use and involvement of differentiated factors within education itself. In this line, active methodologies are part of this set of teaching actions that pursue educational quality and achievement of objectives in a more appropriate way for a student, who is the main protagonist [2].

The new strategies and training methods make no sense if they are not related with students [3] or if they are not connected with their socio-educational reality [4]. Therefore, teaching methodologies must be changed. Teachers should work on the basis that students could design their learning process without giving rise to negative experiences due to poor design or poor planning [5].

All this is possible thanks to the innovative action of teachers to improve the achievement of learning objectives. Because of this, generation of a good class environment is essential to develop these methodologies [6].

### 1.1. Gamification as a Learning Strategy

Gamification arose due to everything mentioned above in the attempt to enhance the mechanisms that facilitate learning and adapt to the interests of students to achieve useful understanding of academic content [7]. The term “gamification” was conceived in the business or marketing area with the idea of customer loyalty [8,9]. This was taken to the school context later on, where teachers and students develop it.

This methodology consists in the application of game dynamics in teaching practice and in the use of game elements or structures in the formal context of the school. With the application of this strategy based on game structure, a dynamic based on their own mechanics is pursued, with a free and voluntary participation in an imaginary world with codes and norms [10,11,12,13].

Gamification improves various psychosocial academic indicators such as students´ motivation, as the activity to be carried out is presented as an attractive challenge for the participants [14,15,16,17,18]. Other benefits found in the literature review are resolution of problems in training activities [19], commitment to the task to be performed [20,21], interest in learning actions that lead to the achievement of didactic objectives [22], attraction to learning that favors the achievement of skills [23], social skills required to interact effectively in group practices [24], and improvement of student behavior and attitude [25].

In addition, gamification is conceived as a way to change the traditional rewards that students are used to, such as grades [26,27], for other, more attractive ones, such as challenges and tests to be carried out, with the freedom to make mistakes and, at the same time, have a follow-up of their own learning. They get badges [28] for trying and effort, not only for achievement, as with the traditional methodology.

### 1.2. Escape Room as a Gamification Methodological Strategy

One of the examples of gamification is the escape room approach. This innovative teaching approach, in addition to implementing the gamification strategy, focuses on learning based on problem solving [29]. Specifically, an escape room consists in the autonomous work of students to resolve a real or a fictitious problem posed by the teacher [30]. Students have to find a solution to it collaboratively, favoring activation and involvement of all [31] in an inclusive process that favors acquisition of skills [32] and results of learning [33].

On the structural level, an escape room is based on games where students are locked in a cabin and must solve challenges, tests, or riddles in order to leave the place where they are [34]. For this, students have a specific limited time [35]. In this regard, establishment of an optimal cooperative work environment and a high degree of student commitment to reach the goal [36] becomes especially relevant. Escape rooms unite aspects of three active learning methodologies, mostly of gamification, due to the game structure and rewards; of flipped learning, as it is performed with a video and instructions, with learning spaces and times; and also of problem-based learning, since students start with an initial problem that they have to solve through what they have learned. As a union of these three methodologies, escape rooms constitute an opportunity to combine positive aspects thereof to improve student learning in playful contexts [37,38].

The design of an escape room is structured in several phases and models. The phases are defined by the teacher who serves as a guide to facilitate the learning process [39] in charge of converting the information obtained by the students into the knowledge that favors learning [40]. The performance models of escape rooms may vary depending on the established test model and may be linear if they follow a fixed sequence; open if the challenges are not linearly related; and multilinear if the previous ones are combined [41].

The students’ work focuses on putting into practice the knowledge acquired during the conceptualization phase of learning with the ultimate goal of “escaping” from a room [42,43]. This implies better acquisition and reinforcement of the learning content, a better activation of students in their learning process [44], and, consequently, greater satisfaction and attitude of the students when developing a more attractive playful formative process [45].

According to the literature, the use of escape rooms in learning spaces is booming [46], reporting positive results [47]. However, there are only few studies regarding such aspects as activation and absence of a negative effect. Specifically, the first construct (activation) focuses on the level of activity that a student achieves while performing the gamified experience. Therefore, activation is understood here as the state of the learner and how he/she faces the task, and his/her personal predisposition to achieve the goals and objectives envisaged by the educational task. Therefore, the concept of activation is understood as active participation of a learner in the learning process by learning something new themselves [48].

This concept can be divided into several categories, such as active, nervous, frantic, or excited. In relation to the absence of a negative effect, it is based on the reflection or perception of students’ negative emotions, such as feeling annoyed, hostile, or frustrated while carrying out the activities or tests proposed by the teacher [49].

To promote adequate levels of activation, the use of training dynamics based on gaming is recommended [50] with the purpose of creating a playful environment where students feel active, see themselves as protagonists and are interested in the tasks to be performed [51]. In addition, to encourage the absence of a negative effect during the teaching and learning process, experts recommend using games. Using an escape room or virtual media through digital applications or video games, students can perform instructional tasks repeatedly without fear of error [15,52].

In this sense, the use of active methodologies becomes a way to transmit and assimilate the didactic contents in a participatory manner benefiting the activation of students in their own learning tasks. Today, students assume the role of builders of their own knowledge with this type of methodologies [53,54], consequently, this contributes to lower anxiety and stress, since they perform tasks at their own pace, autonomously, and individually, according to their personal and cognitive peculiarities. All this is supported by a collaborative work with other classmates. Finally, students actively and without frustration or negative feelings reach the objectives established by the teacher [2].

Given the relevance of the terms offered by experts and due to the lack of information on activation and absence of a negative effect on gamified experiences through escape rooms, this study is presented to analyze these aspects of psychosocial nature in order to offer innovative results of these variables less analysed in the literature. Therefore, this study aims to verify whether the use of escape rooms benefits activation and absence of a negative effect on students.

The educational implications of these methodological approaches are identified here, specifically, the possibilities verified by various studies reported in the previously published impact literature. In addition, playful participation of students in gamified activities results in a higher interest of students in learning, which can be an inexhaustible source of both experiences and positive attitude of regarding the educational event.

This study contributes to the advancement of knowledge in the use of these methodologies, since it implies implementation of different actions. These actions are focused on the analysis of the absence of a negative effect, which is favored by the improvement of academic indicators, such as motivation, cohesion, problem solving, and everything that escape rooms and gamification intrinsically imply. This is also a good starting point to investigate and increase the amount of literature on the state of affairs concerning the scientific educational field.

### 1.3. Development of the Training Experience

**Figure 1:**
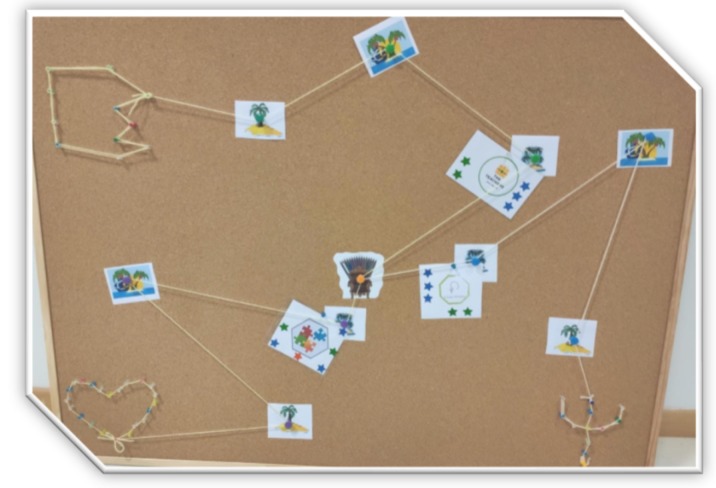
A simplified gamified process map.

In the experimental groups, gamified experience was carried out in 9 sessions. This educational innovation consisted of working the didactic contents by means of an escape room through a thematic map (Figure 1) with gamified elements in order to favor the motivation and interest of the participants. The students had to follow a route whose stations corresponded to the number of sessions. At each of the stations, the students had to carry out different tasks, formative actions, and solve puzzles in order to be able to advance and, at the same time, get badges. Each of the obtained badges served to obtain clues to solve the riddles, as well as to develop the qualifications in the different training tasks.

The final mission of the experience was to get to the pencil throne, where they would compete in a final game for the throne. All groups had the same opportunities to obtain the highest grade and to use the obtained rewards. These rewards could be exchanged for more time to respond, to be able to speak before peers, etc.

On the other hand, an escape room is prepared by the classmates, so that the whole class participates within different groups. The main mission, typical for this activity, is to leave the room having solved all the posed puzzles before the time limit. Finally, the control group followed a classic methodology based on the use of expository classes with small group work carried out within the sessions.

## 2. Materials and Methods

The method followed for the development of this case study is a mixed one. This type of methodology, which is booming within the field of social sciences [55], allows a joint use of qualitative and quantitative methods with the aim of creating an in-depth image about the reality studied [56]. For the quantitative part, the GAMEX (Gameful Experience Scale) scale is used, with the objective of assessing students’ perceptions from different dimensions [49,57]. For the qualitative analysis, the classic process of reducing the information categorization and interpreting the open questions has been done.

**Figure 2:**
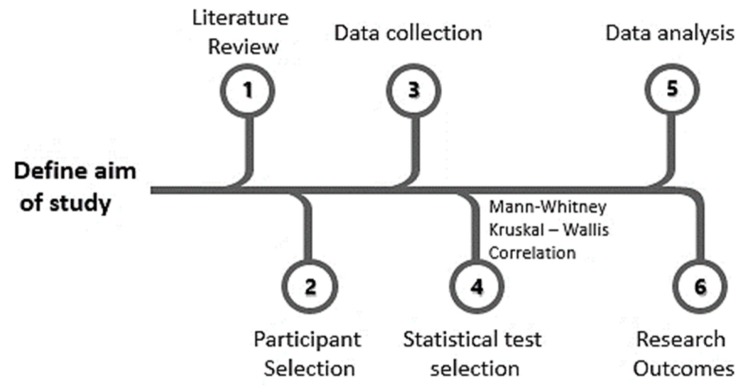
The methodological process followed to develop the study.

Figure 2 shows the process of the methodology followed during the study. First, a literature review was carried out before beginning the collection of the necessary data. Once the characteristics and adequacy of the sample were examined, various non-parametric tests were used to analyze these data. The details of the analyses carried out are specified below.

### 2.1. Aim of This Study

The main aim of this study was to identify how the use of gamification affects the use of educational escape rooms in terms of activation and absence of a negative effect on students.

### 2.2. Study Sample

The sample used for this research was composed of 61 subjects; 21 of them belonged to experimental group 1, 18 belonged to experimental group 2 (to which the gamified teaching methodology was applied through educational escape rooms), and 22 belonged to the control group (to which the traditional teaching and learning methodology was applied). Table 1 shows more details of the sample used. All the students who participated were enrolled in their Master’s studies of different areas in the Autonomous City of Ceuta. All students attended the Innovation in Education class. During the experimentation, the control group followed a teaching methodology of the traditional nature without changes in teaching based on expository classes, in the way to which they are accustomed, while in the experimental groups, the application of a gamified methodology through escape rooms was carried out.

### 2.3. Instrument

The instrument used is the GAMEX (Gameful Experience Scale), the gamified experience scale developed and validated in English and Spanish [49,57]. The scale is composed of six dimensions (1 – Enjoyment; 2 – Absorption; 3 – Creative thinking; 4 – Activation; 5 – Absence of a negative effect; 6 – Dominance) about the experience of the participants in gamified activities or environments.

- Enjoyment (6 items): measures the level of enjoyment of the participants in the activity carried out, with questions whether they liked playing or if they enjoyed doing it and such.
- Absorption (6 items): measures the level of evasion and sense of time of the participants during the performance of the gamified activity, with questions concerning consciousness while playing and time awareness.
- Creative thinking (4 items): measures the level of creativity of the participants during the gamified experience.
- Activation (4 items): measures the level of activity carried out by the participants in the gamified practice.
- Absence of a negative effect (3 items): measures the level of negativity of the participants in their emotions, as well as of frustration during the gamified activity.
- Dominance (4 items): measures the level of confidence of the participants during the development of the gamified experience.

The dimensions analyzed in this case and extracted from the described instrument are the activation and the absence of a negative effect. This is because in the scientific literature, in addition to the importance of the positive affect (emotions), gamification researchers also discuss that negative affective states are relevant for gameful experience [58,59]. The measurement was done with a Likert scale with answers ranging from 1 (totally disagree) to 5 (completely agree).

An open-ended question was also added, in which participants could freely write their opinions or perceptions about the experience in which they participated. It is necessary to clarify that in coherence with the qualitative research approach, these questions could not be raised as a hypothesis to be confirmed, but as a technique to investigate the main interest in depth [60,61].

### 2.4. Data Analyses

For the data analysis, the statistical analysis software for social sciences was used (SPSS version 25 (IBM®, Armonk, NY, USA). After using the equality test of variance (Levene’s test) and the Kolmogorov–Smirnov test, the preliminary results showed that the sample used did not follow the normal distribution. Therefore, for the comparisons made during this study, univariate descriptive tests, the Kruskal–Wallis H test, and the Mann–Whitney test were used. The significance values of the tests followed the classical value in the literature (α = 0.05) [62].

Regarding the qualitative component, a thematic analysis of the answers provided by the participants was carried out. The first manual analysis of the results was done to detect any categories that may arise and had not been previously contemplated. Secondly, the analyses were carried out using the qualitative analysis software Nvivo 10 (QSR International, Melbourne*,* Australia).

## 3. Results

### 3.1. Realiability Analysis

Before performing any inferential analysis to look for statistically significant relationships, it is necessary, to carry out various classic validation tests of the questionnaires. In this case, the three most common values in the scientific literature, Cronbach’s alpha, composite reliability, and the average variance extracted [63,64,65] were analyzed. Table 2 compares the values obtained for the total scale and their comparison with the acceptable results proposed in the scientific literature.

### 3.2. Descriptive Analyzes of Groups

The results obtained for the three groups of subjects analyzed, depending on the dimension analyzed, are shown in this section. It is observed that in the control group, average values were lower than in the other analyzed groups. Among the results shown in Table 3, the activation dimension (2.19 ± 0.66) stands out. Participant members of EG1 are those who obtained better values in the absence of a negative effect dimension (4.35 ± 0.80).

In order to detect the differences between the groups analyzed, the analysis was performed using the Kruskal–Wallis H test. Table 4 shows the results obtained for the two dimensions analyzed. Significant differences were found between the three groups analyzed for the activation dimension (χ^2^ (3) = 10.178, *p* = 0.04) and for the absence of a negative effect (χ^2^ (3) = 26.171, *p* = 0.050).

**Figure 3:**
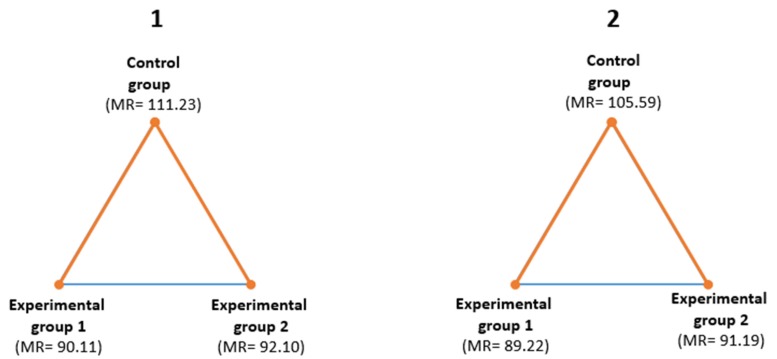
Mann–Whitney U test for the activation dimension (1) and the absence of a negative effect dimension (2). MR: Mean Ranks.

Once the existence of significant differences had been detected, the Mann–Whitney U test was used as a post-hoc test. The results (Figure 3) show the significant differences found (in orange). For the activation dimension (1), the differences were found between the control group (MR = 111.23), experimental group 1 (90.11), and experimental group 2 (MR = 92.10). Similarly, for the absence of a negative effect dimension (2), similar differences were observed between the control group (MR = 105.59), experimental group 1 (MR = 89.22), and experimental group 2 (MR = 91.19).

### 3.3. Qualitative Analysis of the Experience

Analysis of qualitative information was done through a categorization process [66], by which all the information was transcribed to be subsequently grouped by themes or categories. This allowed researchers to detect emerging categories. This process facilitated triangulation of the information, as well as the process of analysis thereof, being the main criterion of validity in the qualitative research (Table 5). This process was carried out using Nvivo 10 (QSR International, Melbourne, Australia).

#### 3.4.1. Motivation

Motivation is positively influenced by the use of a non-traditional methodology such as gamification through educational escape rooms. Thanks to the increased motivation, students are able to obtain better results than they thought they could achieve at first in addition to better expectations and knowledge of their true potential.

“Thanks to the approach of the subject, I think I can achieve better results than I thought I could" (P.3). "I felt able to pass without difficulty in addition to assimilating the contents without having felt anxiety at any time during the process” (P.15).

#### 3.4.2. Group Cohesion

From a group or social perspective, improvement was observed among the students, mainly due to the need to collaborate and communicate with each other in order to obtain various badges. That is, the establishment of common objectives during the sessions led to an increase in-group or social relations.

“Another aspect that I have enjoyed the most was having to work with my colleagues” (P.21). “It has been interesting to have to collaborate to get some of the badges that otherwise I could not have achieved” (P.24).

#### 3.4.3. Engagement

Undoubtedly, the increase in motivation and social or group factors has other effects on student learning, including the increase in their commitment and participation with the subject and the different tasks performed.

“These types of activities have made me want to get the best of myself, especially for my classmates” (P.16). “I could not miss class, I could not leave my classmates alone for activities” (P.6).

## 4. Discussion

The teaching practice in the information and knowledge society has been influenced by the new ways of teaching with a much more playful and attractive educational aspect for students. This is way far from those traditional training actions where only the teacher is the main protagonist and diffuser of knowledge [2]. In the present study, to achieve the formulated objective, an experiment was developed in the university field that allowed verifying its scope.

The results obtained in this study reveal that gamified actions such as escape rooms benefit and enhance academic indicators, such as motivation, group cohesion, commitment, activation, and absence of a negative effect during the teaching and learning process. These results achieved and expressed in the particular context of this study are in line with other studies that preceded this one. These studies reported that training practices using escape rooms improve student motivation [14,15,16,17], group work [24], activation and participation [31,44], as well as commitment to learning tasks [20,36], which has a close psychological relationship with the absence of a negative effect and adequate attitude of students towards the instructive process [25,45].

In this sense, the results show greater activation of students associated with the ability to learn the subject through escape rooms, in direct coincidence with what is reflected in the literature [44]. On the other hand, the findings obtained show a greater attraction for learning than in other cases [31], as well as a more complete training process, as other researchers also conclude [45].

For this reason, thanks to the methodological fusion inherent in escape rooms (flipped learning, gamification, and problem-based learning), an improvement is achieved in the aforementioned academic indicators [2,37,38]. This highlights the importance of active methodologies in training action in order to encourage and enhance student learning [3,7,12,19], as well as to encourage their activation and leadership [31,42,47,50] and to reduce negativity and frustration in their daily work [5,15,25,43,46,49].

## 5. Conclusions

Therefore, as reflected in the literature on the state of the matter and in the findings identified in this study, it is concluded that the use of gamification and, more specifically, the application of escape rooms in learning spaces is beneficial. These methodologies not only promote innovation and revitalization in educational actions carried out by teachers, but also positively affect the psychosocial factors already described.

The prospective that emerges from this research is focused on encouraging the development of innovative practices, such as the use of escape rooms in different educational contexts. In addition to raising awareness, these findings give to the teaching group confidence in gamification and thereby to launch new ways of transmitting knowledge, reducing the traditional practices focused on the simple exposure of content through masterful lessons that fail to attract or motivate students, which cause apathy and the negative effect to increase, leading to failure and, consequently, dropping out of school.

## 6. Limitations and Future Lines of Research

The main limitations of this study and suggestions for future studies are listed in this section. The number of the variables analyzed may not be as large as in other studies, but it is enough for a case study like this one. These variables and their analysis could be studied in a deeper way in the near future. The number of participants can be sufficient for this study and its methodological characteristics, but it could be ideal to perform such a study with more participants. The proposals for a future study are:

- To check if the detected effects occur at lower educational levels.
- To include the social factor in the analysis of gamified experiences.
- To do an in-depth study of the information obtained qualitatively with these dimensions.

## Figures and Tables

**Table 1:** Sample breakdown by group and sex.

Groups	Total	Male	Female
Control group	22	10	12
Experimental group 1	21	10	11
Experimental group 2	18	13	5

**Table 2:** Reliability analysis of the scale and factors used.

Factors	α	α_L_	CR	CR_L_	AVE	AVE_L_
**Activation**	0.89	>0.85	0.864	>0.70	0.615	>0.50
**Absence of a negative effect**	0.88		0.890		0.650	

**Note:** α: Cronbach’s alpha; α_L_: Cronbach’s alpha proposed in the literature; CR: composite reliability; CR_L_: adequate composite reliability proposed in the literature; AVE: average variance extracted; AVE_L_: average variance extracted proposed in the literature.

**Table 3:** Descriptive analysis of the different groups participating in the study.

Factors	EG1		EG 2		CG	
	Mean	σ	Mean	σ	Mean	σ
**Activation**	4.11	0.73	4.12	0.62	2.19	0.66
**Absence of a negative effect**	4.35	0.80	4.25	0.85	3.25	0.69

**Note:** EG1: Experimental group 1; EG 2: Experimental group 2; CG: Control group.

**Table 4:** Results for the Kruskal–Wallis H test for the dimensions analyzed.

Factors	*χ^2^*	*p*
**Activation**	10.178	0.04
**Absence of negative effect**	26.171	0.050

**Table 5:** The categories obtained after interpreting the answers.

	Categories	Definition
Motivation	- Search for better results.	The use of this type of methodologies causes some effect on the motivation of students.
	- Intrinsic motivation/anxiety reduction.	
Group Cohesion	- Collaboration and communication with other colleagues.	Different social capacities can be developed through interaction and cohesion of group work.
	- Establishment of common objectives.	
Engagement	- Effects on activation and participation.	The use of badges has positive effects on students’ commitment to homework.
	- A higher level of commitment is required for tasks.	
